# Stroma: the forgotten cells of innate immune memory

**DOI:** 10.1111/cei.13149

**Published:** 2018-07-10

**Authors:** T. Crowley, C. D. Buckley, A. R. Clark

**Affiliations:** ^1^ Institute of Inflammation and Ageing, College of Medical and Dental Sciences University of Birmingham Birmingham, UK; ^2^ Kennedy Institute of Rheumatology, University of Oxford, Oxford, UK University of Oxford Oxford UK

**Keywords:** endothelial cell, fibroblast, inflammation, innate immune memory, stromal memory

## Abstract

All organisms are exposed constantly to a variety of infectious and injurious stimuli. These induce inflammatory responses tailored to the threat posed. While the innate immune system is the front line of response to each stimulant, it has been considered traditionally to lack memory, acting in a generic fashion until the adaptive immune arm can take over. This outmoded simplification of the roles of innate and acquired arms of the immune system has been challenged by evidence of myeloid cells altering their response to subsequent encounters based on earlier exposure. This concept of ‘innate immune memory’ has been known for nearly a century, and is accepted among myeloid biologists. In recent years other innate immune cells, such as natural killer cells, have been shown to display memory, suggesting that innate immune memory is a trait common to several cell types. During the last 30 years, evidence has slowly accumulated in favour of not only haematopoietic cells, but also stromal cells, being imbued with memory following inflammatory episodes. A recent publication showing this also to be true in epithelial cells suggests innate immune memory to be widespread, if under‐appreciated, in non‐haematopoietic cells. In this review, we will examine the evidence supporting the existence of innate immune memory in stromal cells. We will also discuss the ramifications of memory in long‐lived tissue‐resident cells. Finally, we will pose questions we feel to be important in the understanding of these forgotten cells in the field of innate memory.

## Introduction

In an immune setting, the term ‘memory’ evokes vaccines, memory T cells and the antibody response. Memory in mammals is not, however, solely the remit of lymphocytes. The field of immunology is changing, and evidence in both arms of the immune system is breaking down old concepts of innate and adaptive roles.

Inflammatory memory is an ancient characteristic of innate immune cells. Vertebrate–invertebrate divergence preceded the appearance of adaptive immunity, and yet evidence in invertebrates as disparate as meal worm beetles and shrimp, *Drosophila* and lower metazoans all points towards inflammatory responses altered by prior exposure to infectious agents (reviewed in 1).

Innate immune memory in mammals was first observed during the early 20th century. The bacillus Calmette–Guerin (BCG) vaccine was found to save more children than died of tuberculosis, indicating an off‐target effect 2. This work has progressed over decades to illustrate innate memory in a range of experiments studying mammalian defence against secondary pathogens. Crucially, studies in severe combined immune deficiency (SCID) mice have shown the BCG off‐target protection to be driven by innate – not adaptive – memory 3, 4. Such research has focused mainly on myeloid cells, with other haematopoietic cell types also attributed to innate memory in recent years (reviewed in 5 and 6).

Despite the abundance of research on innate memory, stroma is still largely overlooked when considering repeat challenges in immune or inflammatory episodes. Indeed, the recent discovery of epithelial innate memory 7 prompted a *Nature* news and views article, which stated: ‘This is the first identification of inflammatory memory in a non‐immune cell’ 8. It is clear that stromal memory is not well‐known or appreciated.

Given the recent resurgence of interest in stromal memory responses, and the latest observations showing that haematopoietic, epithelial and stromal cell types all alter their responses based on previous insults, we believe it is time to discuss the field of stromal innate memory. In this review, we will collate three decades of stromal memory research, discuss its strengths and unanswered questions and suggest key avenues of enquiry. Innate memory is proving ever more important to our understanding of inflammation biology, and it is time that stroma, the forgotten cells of the memory field, were remembered.

Because some of the important terms in this review may be subject to different interpretations, we will begin with some definitions. The ‘stroma’ comprises ubiquitous structural cells, providing extracellular matrix (ECM) and support functions in every organ. In this review we take ‘stromal cells’ to exclude cells of haematopoietic origin; for example, tissue‐resident macrophages. Macrophages and other myeloid cells are discussed below merely by way of introduction to the concept of memory. To our knowledge, the vast majority of the research relevant to stromal memory has been performed on fibroblasts and endothelial cells. We therefore focus on these cells, but we consider it very likely that memory also exists in other stromal cell types. We define ‘memory’ as a state of altered responsiveness that does not depend upon the continued presence of the stimulus by which it is evoked. A key characteristic of this form of memory is that it outlasts the primary signalling response of the cell. In the experiments discussed below memory lasts from days to weeks, or even longer. There will clearly be some overlap between memory and differentiation, for example in a few experimental designs discussed below, where stromal cells are exposed to persistent inflammatory stimulation rather than being stimulated, rested, then stimulated again. As far as this review is concerned, we define ‘inflammatory memory’ as a change in the capacity of stromal cells to respond to inflammatory stimuli, as opposed to an inflammation‐induced change in steady‐state gene expression.

## Stroma in inflammation

During the last three decades, our understanding and appreciation of the roles played by stromal cells in healthy and pathological inflammation has increased exponentially. Two well‐studied stromal cells [endothelial cells (EC) and fibroblasts] are described below.

EC line the blood and lymph vessels, and thus have an important role in inflammation. Both host and foreign cells must interact with EC in order to extravasate into tissue. Their release of chemokines recruits leucocytes to a site of infection or injury, and their expression of adhesion molecules facilitates adhesion, rolling and diapedesis of circulating cells (reviewed in 9).

Fibroblasts are a major source of ECM and play organ‐specific roles, such as lubricin production in the joint and lymphocyte support in lymphoid organs. They act as immune sentinels, with some capacity for phagocytosis 10 and antigen presentation 11. They also release chemokines to recruit leucocytes and cytokines to modulate the nearby EC 12. The fibroblast influence on EC adhesion molecule expression has profound effects on leucocyte extravasation. In healthy inflammation fibroblasts limit extravasation through EC, while fibroblasts from inflamed joints promote increase leucocyte extravasation 13, 14.

**Figure 1:**
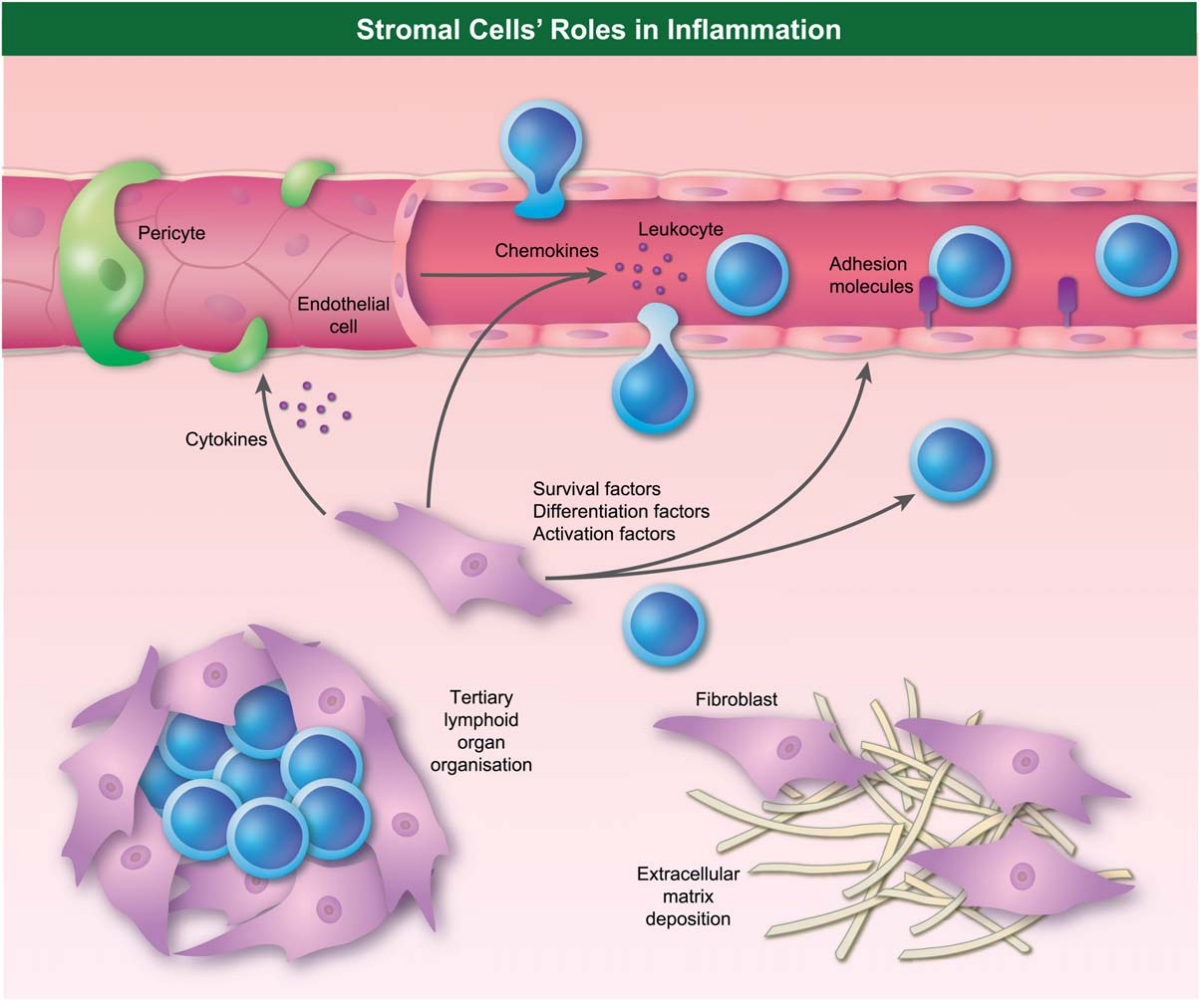
Roles of stromal cells in inflammation. Illustration of the functions stromal cells use to influence the fate of an inflammatory episode. Secreted and presented mediators are illustrated, as are as key organizational roles.

Once leucocytes extravasate into tissue, fibroblasts influence their behaviour via the regulation of survival, activation, differentiation and retention 15, 16. It is therefore not surprising that pathologically altered fibroblasts are integral to several chronic inflammatory diseases. The roles of stromal cells in inflammation are illustrated briefly in Fig. 1. Due to the number of roles played, understanding how these cells alter their behaviour after an initial inflammatory episode could be crucial to our understanding of human pathology.

### Myeloid innate memory

Monocyte/macrophages are innate cells of the myeloid lineage, which act as immune sentinels, phagocytes of pathogens and cellular debris and as professional antigen presenters. In this sense, they are often recognized as the link between innate and adaptive immunity. This is also true in a temporal sense, as many studies have found that monocytes infiltrate tissue after neutrophils but before lymphocytes 17, 18. Others also found that the phagocytosis of apoptotic neutrophils by macrophages (known as efferocytosis) is a key point in progressing from the acute to the adaptive phase of inflammation (reviewed by 19).

As an intermediary between the two arms of our immune system it is perhaps not surprising that, of all innate cells, macrophages should have memory. This may take either positive or negative forms, in that the response to a second challenge may be either enhanced or diminished by memory of a prior challenge. Both short‐ and long‐term forms of myeloid cell memory have been described. Long‐lived myeloid cell memory that underpins the beneficial off‐target effects of vaccines such as BCG has been coined ‘trained immunity’ by Netea and colleagues 1. This phenomenon is exhibited only after the cells have been educated by an initial infection, lasts for several weeks or even months after the initial infection, and induces an augmented inflammatory response to second infection. The long‐term alteration is driven by epigenetic modifications, which are described in more detail later. Trained immunity has been studied in a wide range of primary and secondary challenges, and particular attention has been paid to the BCG vaccine and fungal products (β glucan) or organisms (*Candida albicans*) as providing wide‐scale protection against a range of secondary agents. This protection takes the form of lower mortality rates and better pathogen clearance 4.

The other form of memory is a transient reduction of the inflammatory response. In 1947 bacterial substances were found to have diminishing pyrogenic effects in rabbits receiving successive doses 20. In 1965, this was furthered by research showing that rabbits could be saved from death by a lethal dose of liposaccharide (LPS) if they had previously received a sublethal dose 21. This phenomenon was termed ‘endotoxin tolerance’, and was attributed to macrophages some time later. While tolerance implies no effect of endotoxin on the macrophage, this is an over‐simplification. It is true that successive doses of endotoxin abrogate the expression of proinflammatory mediators but, contrastingly, increase expression of genes related to phagocytosis 22. This results in a phenotype which avoids tissue damage while still facilitating removal of infectious agents. It is therefore important to distinguish it from ‘immune paralysis’: the state of disorganized immune response seen after trauma or burns, and a contributing factor to mortality in sepsis patients 23. Endotoxin tolerance is therefore a sophisticated state of altered responsiveness, rather than simply an ‘off switch’ for macrophages.

### Fibroblast inflammatory memory

The study of fibroblast memory goes back to 1992 24. Early papers arose from discoveries pertaining to modulation of interleukin (IL)‐8 expression by interferon (IFN). After the discovery that IFN‐β or ‐γ would reduce tumour necrosis factor (TNF)‐α‐induced IL‐8 expression 25, a series of publications examined the effect of IFN on endogenous or exogenous induction of IL‐8, including the consequences of challenging fibroblasts with one stimulus and then another 24, 26, 27, 28. Thus, the field was born.

The earlier years of the new millennium saw no new publications on the subject, and it appeared forgotten. In the interim, appreciation of fibroblast involvement in chronic inflammatory diseases was continuing to grow. With the greater understanding of stromal epigenetic alterations resulting in long‐term increases or decreases of chromatin accessibility, authors began to suggest that a ‘fibroblast inflammatory memory’ may be involved in the perpetuation 29, 30 or re‐ignition 31, of inflammatory conditions.

At the same time, the primary publications on fibroblast memory were re‐emerging. In 2009 gingival fibroblasts were shown to maintain their inflammatory response to re‐challenge with LPS, whereas macrophages became refractory 32. In 2011, however, gingival fibroblasts were shown to be capable of tolerance, but in a gene‐specific fashion 33. A recent spate of publications 34, 35, 36, 37, 38 has advanced our understanding further. Sohn *et al*. displayed inflammatory memory of T cell chemoattractants in fibroblast‐like synoviocytes (FLS) derived from patients with rheumatoid arthritis (RA) 39. Our own work built upon these findings by displaying positive memory to be shared by FLS from both inflamed and non‐inflamed joints 37. While both papers showed gene‐specificity of memory in response to endogenous stimuli, the latter also displayed trophism, showing that FLS had memory while dermal fibroblasts did not. These findings suggest that inflammatory memory can be a property of the site of origin of fibroblasts rather than disease state.

Recently, Klein *et al*. also demonstrated the tropism of fibroblast memory and its gene‐specificity 38. Repeat challenges with LPS induced a refractory state seen only in fibroblasts of certain sites, and only in genes linked to the anti‐viral response. Dakin *et al*. showed that tendon fibroblasts from chronic tendinopathy patients mounted stronger inflammatory responses *in vitro* than those of healthy tendons, thus demonstrating the longevity of innate immune memory in fibroblasts 35, 36. They also discovered that while intracellular signalling diminished over time after removal of stimulus, surface markers of fibroblast activation such as podoplanin (PDPN) and vascular adhesion molecule‐1 (VCAM‐1) were maintained at high levels. This suggests that memory in stromal cells does not necessarily manifest as continued inflammatory secretion, but rather as an altered state of readiness with increased activation receptors 36 and altered chromatin accessibility 38, 39. The altered expression of some fibroblast cell surface markers at sites of chronic inflammation could be interpreted as a differentiation phenomenon. Alternatively, it could reflect expansion of a fibroblast subpopulation that responds differently to inflammatory challenge. These interpretations cannot be resolved without deeper understanding of the molecular mechanisms involved.

### Endothelial cell inflammatory memory

Research into the concept of endothelial cell memory is newer than that of fibroblast memory, but a range of challenges have been examined. This field dates at least to 2002 40, and publications are increasing in frequency. As seen in fibroblast memory, some authors have not found an altered response to second challenge 41, while others have shown both positive and negative memory responses indicative of a complex interplay of stimuli and cellular pathways. The examination of cross‐stimulation (initial and secondary stimuli inducing different pathways) has led to the conclusion that some mechanisms of innate memory are shared with the myeloid lineage 42, but not all observations match, suggesting cell‐ and stimulus‐specific pathways.

The anatomical site of origin and inflammatory stimulus have been shown to dictate EC memory responses, and the vessel architecture has also been demonstrated as important. Shear stress (pressure exerted by blood flow on the vessel walls) is higher in straight vessel sections than arches or junctions. Work from the Evans laboratory has shown that EC under low shear stress have constitutively active c‐Jun N‐terminal kinase (JNK) 43, which is suppressed by dual specificity phosphatase‐1 (DUSP‐1) in areas of high shear stress 44. This high level of JNK activity correlates with VCAM‐1 expression and thus the ability to facilitate leucocyte adhesion. The authors described JNK as ‘priming’ EC for inflammation, and pointed out that primed areas were atheroprone, while areas with high shear stress and suppressed JNK activity were atheroresistant.

Studies have confirmed the relevance of EC memory in both infectious and non‐infectious inflammatory contexts. Prior infection by *Schistosoma mansoni* increases leucocyte adherence through the endothelial layer 45. Others have shown that EC exposed to circulating contributors to atherosclerosis have augmented inflammatory responses to subsequent challenges by exogenous or endogenous stimuli 40, 46. In the examples above, altered EC responses were driven by persistent exposure to inflammatory stimuli. Whether such changes should be considered as memory or differentiation is arguably a question of semantics.

In 2008, Zemani *et al*. found that EC progenitors performed better as therapeutic cells in models of ischaemic injury if they were primed *ex vivo* with C‐X‐C motif chemokine ligand 12 (CXCL12) before donation 47. Similarly, in 2016 Stark *et al*. found the endotoxin tolerance seen in EC could be manipulated. An initial challenge with LPS induced an inflammatory response, which was followed by non‐responsiveness to subsequent LPS challenges. If, however, monophosphoryl lipid A (MPLA) was used as the initial stimulus, the non‐responsiveness to LPS was achieved without an initial inflammatory event 48. This suggests that EC memory may be amenable to therapeutic manipulation. Thus, there is an obvious need to understand endothelial memory at basic science and clinical levels.

## Mechanisms underpinning innate memory

The mechanisms behind innate memory have been investigated intensively in the myeloid lineage. The effects of Toll‐like receptor (TLR)‐4 signalling in particular have received most attention, and have been reviewed comprehensively 49. For this review, it suffices to say that TLR‐4 signals through the myeloid‐differentiation primary response protein 88 (MyD88) and Toll/IL‐1R (TIR)‐domain‐containing adapter‐inducing IFN‐β (TRIF) pathways. MyD88 induces proinflammatory cytokine expression through pathways such as that of nuclear factor kappa B (NF‐κB). TRIF induces the interferon response and thus the production of IFN response factors (IRF) and anti‐viral effectors. These pathways are well established and reviewed by 50.

### Negative feedback signalling

The proinflammatory TLR‐4 response is, as with all healthy inflammatory episodes, curtailed by in built feedback loops 51, 52. Both the MyD88 and TRIF pathways induce the expression of negative regulators. These may inhibit TLR‐4 signalling at levels distal, intermediate or proximal to gene expression. IL‐1 receptor‐associated kinase (IRAK) M and A20, for example, inhibit, respectively, the release of IRAKs and TNF receptor‐associated factor (TRAF)6 from MyD88, and the actions of TRAF6 and TRIF 53, 54. Conversely, SH‐2 containing inositol 5' polyphosphatase (SHIP) 1 inhibits inhibitor of κB kinase (IKK) activity downstream of MyD88 and IRF3 downstream of TRIF 55. Finally, the non‐canonical NF‐κB p50 homodimer can inhibit canonical NF‐κB binding to promoters, presumably by steric hindrance 56.

Two studies of fibroblasts are useful to note here. The first is that of Lee *et al*. 57, who showed that rheumatoid arthritis RA‐derived FLS maintained an inflammatory response to TNF‐α up to at least 4 days after initial challenge. The authors also displayed FLS expression of several of the negative regulators to be negligible compared to expression in macrophages. The second is that of Ara *et al*. 32, who found that neither *Escherichia coli* LPS nor *Porphyromonas gingivalis* LPS (TLR‐4 and TLR‐2 agonists, respectively) could induce tolerance in gingival fibroblasts. They also showed that human gingival fibroblasts lack expression of classic negative regulators. During myeloid endotoxin tolerance, negative regulators are up‐regulated, leading to disrupted signalling through both pathways. While the above fibroblast studies showed a deficiency in negative regulators, neither showed explicitly that this was the reason for propagation of the inflammatory response. Future studies are necessary to link these observations with alterations in memory responses, as has been confirmed in myeloid cells 58.

### IFNs

IFNs are induced in response to infectious agents, and also in response to tumour cells and proinflammatory cytokines. Their role in innate memory appears multi‐faceted. IFN‐γ, for example, can rescue endotoxin tolerant macrophages from their refractory state by opening condensed chromatin 59. Conversely, IFN‐β can induce tolerance of a subset of genes in fibroblasts 24, 33. Even in studies of the same cell type (i.e. fibroblasts), IFNs were shown to have both inhibiting 24, 26, 28 and augmenting 26, 28 roles. This disparity was not due to the IFN, but rather to the timing of its administration. Sohn and colleagues found that IFN‐α, ‐β and ‐γ could all induce augmented responses from TNF‐α‐primed FLS 34. This not only confirms that different IFNs may have redundant roles in innate immune memory, but also provides evidence that they may act as the initial or secondary challenge.

Koch *et al*. developed a theory based on cross‐priming EC with TLR agonists 42. They suggested that initial stimulation induced an increase in IRF‐7 accumulation, facilitating a greater response to subsequent challenges. Such a theory may be supported by the finding that FLS have a TNF‐α‐induced IFN‐β autocrine feedback loop which increases chemokine release 60. If both mechanisms were conserved across FLS and EC, one could envisage the initial stimulation providing a large IRF pool, leading to a greater cellular response to autocrine IFN feedback in the second response. This model relies upon uniting mechanisms from different cell types. Even among fibroblasts of different origins this may be inappropriate, as synovial fibroblasts release IFN‐β 60, while those from the gingiva do not express it 33. The effects of trophism, and of different experimental designs with different cell types, stimuli and readouts, means that three decades of research have yet to provide a clear model for the role of interferons in stromal memory.

### NF‐κB

The NF‐κB pathway is well known for its involvement in the expression of proinflammatory genes. In macrophage endotoxin tolerance, the canonical NF‐κB pathway is refractory. Its inhibition is induced by the increase in inhibitory p50 homodimers at the loci of proinflammatory genes 56. There is also increased repression of the IKK complex. Due to this, the abundance of Iκ‐Bα is higher in tolerized than naive cells 61, and it has also been reported to replenish its cytoplasmic pool faster in the second challenge compared to the first 58, 59.

Studies have shown that repeated TLR‐4 engagement leads to repression of the NF‐κB pathway in endothelial cells 42, 46. This included decreased activation of the IKK complex and concomitant reduction in NF‐κB‐dependent cytokines. In direct contradiction, Wang *et al*. found that the second challenge with TLR‐4 agonist increased EC cytokine production, and this correlated with increased NF‐κB pathway activity 41. Both studies used human umbilical vein ECs and *E. coli* LPS for their experiments, so the explanation for the disparity is unclear. What is clear, however, is that the action of the NF‐κB pathway during the memory response correlated with the increase or decrease of inflammatory mediators elicited by the second challenge. There is also evidence for NF‐κB‐dependent altered responses in fibroblast memory. In fibroblasts stimulated with TNF‐α for 24 h then rested for 24 h, both NF‐κB activity and IL‐6 expression subsided to basal levels during the rest period. Restimulation of the rested cells caused more prolonged NF‐κB activation, and increased expression of IL‐6 that was dependent upon NF‐κB. At least in this case, the inflammatory memory of fibroblasts is not simply persistence of cell activation, but reflects priming of the NF‐κB signalling pathway for an augmented response 37. The Kalliolias group showed that TNF‐α‐treated RA FLS continued to express inflammatory mediators for up to 4 days in a manner dependent upon sustained NF‐κB activity 56. They showed later that NF‐κB was also required for priming of these TNF‐α‐exposed cells to respond more strongly to IFN stimulation 34.

### Chromatin access and epigenetics

Epigenetic modification is generally regarded as key to the innate memory seen in macrophages. Trained immunity induces an augmented response to second challenge weeks or even months after the initial infection. Changes on such a time‐scale are likely to be epigenetic and, indeed, evidence abounds for such a theory. Permissive modifications have been described by several groups 4, 62.

Endotoxin tolerance is a memory response of a much more short‐term nature. While previous examples show evidence for upstream mediators, the study of epigenetics has transferred well to the tolerance field. Examples of this include the length of endotoxin tolerance in macrophages, which has been shown to be from 24–48 to 5 days in duration 22, 63. Medzhitov and colleagues showed that macrophage endotoxin tolerance was induced by gene‐specific transcriptional repression, occurring at the level of chromatin condensation 22. This is in accordance with findings of other groups 59, 64.

The chromatin landscape in stromal memory is receiving increasing attention. When EC were primed with TLR‐3 agonist, the CXCL10 induction by subsequent TLR‐4 agonist was augmented and this was reduced by inhibiting histone deacetylases 42. This suggests that the induction of chromatin opening is part of the augmented stromal memory response. Studies in fibroblasts have also suggested epigenetic changes 29.

More explicit evidence came from Sohn *et al*., who showed that chronic exposure of RA FLS to TNF‐α led to decreased histone H4 levels, increased H4 acetylation and increased NF‐κB and PolII occupancy at the promoter of genes which could be augmented subsequently upon rechallenge. Blocking TNF‐α with infliximab for 24 h before the rechallenge did not reduce the augmented response, suggesting that a permissive state had already been established. This may correlate well with the latent enhancers found to have prolonged transcription factor occupancy in trained macrophages 65. The recent publication by Klein *et al*. also showed the chromatin landscape to determine which genes became tolerant in macrophages and synovial fibroblasts derived from different anatomical sites 38. Genes that could not be tolerized had similar epigenetic markers in the first and second responses to LPS. Those that were refractory in the second challenge displayed decreases in histone markers associated with accessibility, thus showing that epigenetic silencing contributes to the control of which genes display innate immune memory profiles.

**Figure 2:**
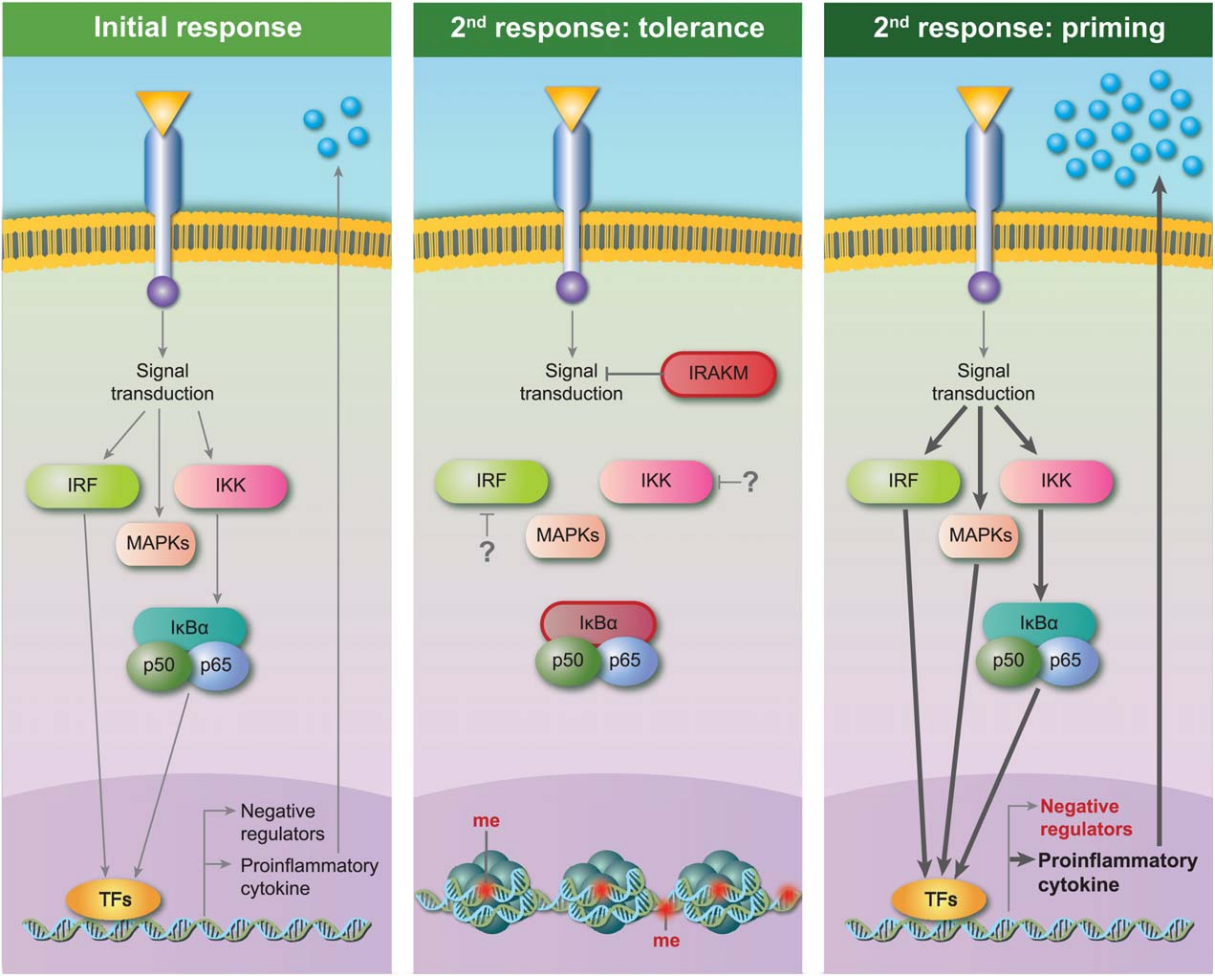
Mechanistic responses to first and second response to stimulation. Simplified stromal cell response to initial response (left), and abrogated (centre) or augmented (right) memory response to second challenge.

A generic example of the signalling events known to occur in stromal cells undergoing tolerance or augmented responses is illustrated in Fig. 2.

## Role of stromal memory in infection and chronic inflammatory disease

We are well aware of the deleterious nature of flawed adaptive immunology. The number and variety of autoantibodies contributing to a large range of autoimmune diseases displays the danger of what is ostensibly beneficial in infection.

Endotoxin tolerance reduces the inflammatory response of myeloid cells to infectious triggers and thus preserves the host's life. Very low doses of LPS, however, do not induce tolerance, but rather a chronically inflamed state 66. In this sense, it is possible that low‐dose stimulants actually prime the host to augmented second responses. The augmented second response to infection is well reported *in vivo* and in macrophages 5. The ‘red alert’ of innate immune cells may induce heightened responses to infectious triggers, but also similarly to endogenous factors.

The concept of endogenous factors priming the response to damage‐associated molecular patterns (DAMPs) is established in the stromal memory field. Homocysteine (HCy) is a circulating amino acid used as a clinical marker for atherosclerosis risk. It has been shown to synergize with TNF‐α to induce stronger activation of EC 40. The authors hypothesized that HCy may prime EC for augmented responses to damage. While not tested explicitly in the publication, this was proved by another group, who showed that concentrations of HCy only slightly higher than normal physiological range could prime aortic EC to an augmented inflammatory response to LPS or thrombin [an exogenous pathogen‐associated molecular pattern (PAMP) and an endogenous DAMP, respectively] 48.

**Figure 3:**
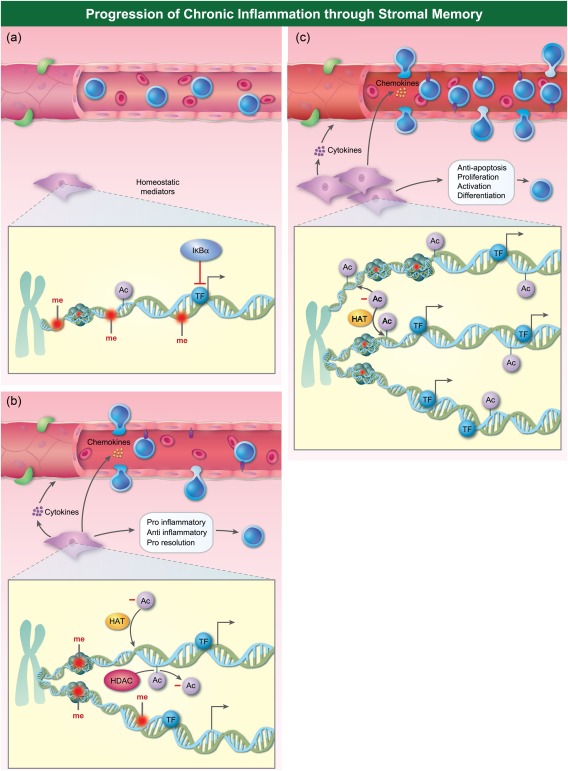
Progression of chronic inflammation through stromal memory. The increasingly inflamed stromal microenvironment during challenge with endogenous mediators is illustrated. (a) The stromal compartment at rest. (b) The inflammatory response of stromal cells leads to an inflamed microenvironment, but negative regulatory and pro‐resolution pathways ensure timely resolution. (c) Continued challenges lead to increased endothelial cell adhesion molecule expression and chemokine expression. Fibroblasts also increase cytokine and chemokine release, inducing inflammatory responses from both endothelial cells and leukocytes. Magnified image illustrates the changes at the chromatin level.

The role of EC as inflammatory contributors to atherosclerosis is well established 44, 67, and the role of myeloid trained immunity in the disease is also gaining attention (reviewed in 68, 69. These publications and others 34, 37, 40, 48, 70 acknowledge the dangers of innate memory facilitating over‐aggressive inflammatory responses based on priming by endogenous mediators. DAMPs and other host products inducing this high alert state may lead to chronic inflammatory conditions because they will tend to favour sustained inflammatory responses rather than resolution of inflammation. In some of these examples, whether augmented inflammatory responses are dependent upon continued presence of the ‘priming’ agent remains to be tested formally. The effects of stromal memory responses to endogenous ligands is shown in Fig. 3.

Research into fibroblast memory also suggests a pathological role. Our own research into non‐inflamed and RA synovial fibroblasts suggests that the augmented proinflammatory response is inherent to the joint 37. This may help to explain the capacity of antibodies against widely expressed antigens to drive joint inflammation. Recent research from Oxford University suggested that chronic tendinopathy was due to an initial (and supposedly resolved) insult, which led nonetheless to fibroblasts acquiring a fibrotic and hyper‐responsive phenotype 35, 36. As remarked above, the altered fibroblast phenotype may be explained in part by differentiation or expansion of a fibroblast subpopulation.

Two publications have shown healthy skin fibroblasts to be incapable of the augmented response 32, 37. In contrast, unpublished work from our department suggests skin fibroblasts from psoriatic plaques pathologically acquire augmented inflammatory responses to rechallenge (i.e. positive memory; manuscript in preparation). This concept of fibroblasts in a chronically inflamed setting acquiring inflammatory memory appears to be supported by fibroblasts from other tissues we have studied, and particularly gingival fibroblasts. These cells have inflammatory memory when isolated from periodontitis patients (manuscript in preparation), but not from healthy patients 32, 33.

The concept of changes in fibroblasts contributing to chronic joint inflammation is well established 71, 72. The perturbation of surface markers leading to site‐specific leucocyte recruitment (the stromal postcode) in chronic inflammation was reviewed more than 10 years ago 73. The idea that fibroblast memory may be inherent in some sites, but gained aberrantly in others, is therefore not an unusual notion.

## The clinical significance of stromal memory

A host of publications have recognized the modulating influence of prior inflammatory events 68, 74, 75, 76, 77. This will obviously due be partly to the adaptive immune response, but as this review has shown, the effect on other cell types, and so the tissue or even organism as a whole, are not to be dismissed. Didierlaurent and colleagues discussed the effects of repeat lung infection 74, while Dakin *et al*. hypothesized 31 and later confirmed 36 that an acute injury event may prepare tendons for chronic tendinopathy and fibrosis upon a second insult. Such concepts show a clear clinical relevance to repeat challenge of innate cells.

Vaccines have proved beyond doubt the clinical benefit of harnessing adaptive immunity. In the same sense, macrophage innate memory is host‐protective in various infectious scenarios. Trained immunity protects the host by putting macrophages (and NK cells) on high alert for subsequent infections. Conversely, endotoxin tolerance reduces inflammatory secretions but maintains phagocytic function to defend the host from cytokine storm.

These two sides of innate memory present an attractive therapeutic opportunity. Mihai and van der Meer have expounded the theory of an ‘innate vaccine’ in order to harness trained immunity 5. This has, in a way, already been invented in the form of the BCG vaccine mentioned earlier, and trials to explicitly test its validity as an infection non‐specific vaccine have been conducted 78. Importantly, the innate vaccine may also be used to defend the host against non‐infectious conditions such as autoinflammatory and allergic diseases 79. Harnessing the short‐term memory of macrophages has yet to come to fruition, and the similar (although distinct) ‘immune paralysis’ of sepsis patients continues to play a deleterious role 80.

Although the field of stromal memory is still relatively understudied, clinical relevance has already been evidenced. As stated earlier, *ex‐vivo* challenging of EC progenitors with CXCL12 induced greater homing to activated EC and greater tube formation 45. The authors suggested that this priming dose would improve efficiency of cell therapy following ischaemic disease. In 2016, Stark *et al*. showed that *in‐vitro* endotoxin tolerance could be achieved in EC by stimulating with MPLA rather than LPS 46. This would render EC refractory to infectious triggers of inflammatory responses without the inflammation induced by the first LPS challenge. Such a mechanism would be of benefit in the setting of chronic inflammatory disease, wherein the EC themselves, or the underlying inflamed tissue, induce pathological recruitment to, and extravasation through, the endothelium. A possible example is periodontitis, in which microbiota and host immune responses conspire to produce the most common chronic inflammatory disease in the world 81. Zaric *et al*. showed that induction of tolerance could be achieved in gingival fibroblasts if IFN‐β was provided 33. Such a reduction in inflammatory response may provide a break in the inflammatory cycle and facilitate the induction of resolution. While targeting circulating cells is always easier than a specific resident population, this latter option is progressing, with improved targeted drug delivery making the targeting of tissue‐resident stromal cells a real possibility 82.

## Future questions

The increased interest in this field during the last few years has shed light upon several facets of stromal memory. It is, however, still a field in its in infancy. The aspects we feel to be most pressing are as follows.

Although *in‐vitro* challenge and rechallenge experiments can shed light upon mechanisms of stromal cell memory, they model the situation poorly *in vivo*. Ground‐breaking experiments by Müller‐Ladner and colleagues demonstrated that RA synovial fibroblasts could be maintained in tissue culture without losing their erosive properties in an *in‐vivo* model of cartilage degradation 83. It has been argued 70 that prolonged exposure of synovial fibroblasts to an inflammatory environment engenders lasting epigenetic changes, for example at the level of DNA methylation or micro‐RNA expression, which strengthen responses to proinflammatory stimuli. However, the acquisition of the aggressive phenotype is not yet well understood. We need better integration between *in‐vitro* approaches and elegant *in‐vivo* experiments of this kind. This may require the use of irradiated chimeras, or even models deficient in immune cells.

The role played by metabolism is currently not known. The field of immunometabolism has expanded rapidly in recent years, and inflammatory triggers are known to alter metabolic processes in both haematopoietic and stromal cells 84. Further, chronic inflammatory diseases are known to correlate with altered cellular metabolic profiles 85, 86. A precedent for the involvement of metabolism in innate memory already exists, as the Netea group have shown that monocyte training by prior challenge involved a shift towards glycolytic metabolism both *in vitro* and *in vivo* 62, 87. Given the link these papers made between innate memory, epigenetics and metabolism, the ability to understand and manipulate stromal memory could depend upon a greater appreciation of this axis.

As described earlier, there is disparity between *in‐vitro* repeat stimulation and real‐world inflammatory pressures. Therefore, although some features of inflammatory memory of stromal cells have been described, the adaptive value of the phenomenon remains unclear. Its gene‐specific nature suggests that it may be a mechanism to fine‐tune the recruitment of leucocytes during the evolution of an inflammatory episode, or to modify inflammation‐induced recruitment to sites that have previously experienced inflammation.

The contribution to chronic inflammatory disease is also yet to be defined clearly. Why is it that stromal memory differs according to site of origin 37, 38, 42, 46? Why may it also differ between cells from healthy and chronically inflamed tissue? Stromal positional identity is long established, and shows at both epigenetic 88, 89 and functional levels 13, 90, 91. If some sites (such as the joint) are more inclined to stromal memory, does this contribute to the likelihood of chronic inflammation in that site? Such knowledge could provide a powerful new prophylactic tool in those with susceptibility alleles.

Finally, if cells from chronically inflamed tissue have memory, is it part of the cause for inflammation or is it acquired in the course of inflammation? If the former, we have a new susceptibility marker which may be more tissue and disease‐specific than most of the genome‐wide association study (GWAS) hits (which are often shared among many diseases). If the latter, this provides a new therapeutic target which could be applied to any chronic inflammatory disease where memory is acquired. Such a therapeutic would have the dual benefit of targeting more than one disease, and of leaving the patient immunocompetent.

## Conclusions

Innate memory in humans was recognized nearly a century ago, and for years was seen as the prerogative of myeloid cells. Innate memory, whether in the guise of endotoxin tolerance or trained immunity, is host protective. The former protects from tissue damage and cytokine storm, while the latter provides a generic augmented response to future infection. Recently non‐myeloid lineages were also recognized to exhibit memory, and stromal memory is starting to receive the attention it deserves.

The roles of stromal cells in inflammation are manifold, and crucial in inflammatory responses to both infection and injury. The field of stromal memory is still in its infancy, and yet already promises to reshape our understanding of stromal cells’ roles in inflammatory episodes. Of the evidence presented herein, some suggest that stromal memory may be adaptive, and others deleterious. Some suggest a double‐edged sword, protecting against infection at the risk of autoinflammatory conditions. The data are varied, but the consensus in the field is that this under‐investigated mechanism will undoubtedly influence the nature, magnitude and duration of inflammatory episodes. Stromal memory, therefore, is well worth remembering.

## Disclosure

None declared.

## References

[1] Netea MG , Quintin J , van der Meer JW. Trained immunity: a memory for innate host defense. Cell Host Microbe 2011; 9:355–61. 21575907 10.1016/j.chom.2011.04.006

[2] Naeslund C. Experience of vaccination with BCG in the province of Norrbotten (Sweden). Tuberculosis Review 1931; 12:617–36.

[3] Kleinnijenhuis J , Quintin J , Preijers F *et al* Bacille Calmette–Guerin induces NOD2‐dependent nonspecific protection from reinfection via epigenetic reprogramming of monocytes. Proc Natl Acad Sci USA 2012; 109:17537–42. 22988082 10.1073/pnas.1202870109 PMC3491454

[4] Quintin J , Saeed S , Martens JH *et al* *Candida albicans* infection affords protection against reinfection via functional reprogramming of monocytes. Cell Host Microbe 2012; 12:223–32. 22901542 10.1016/j.chom.2012.06.006 PMC3864037

[5] Netea MG , van der Meer JW. Trained immunity: an ancient way of remembering. Cell Host Microbe 2017; 21:297–300. 28279335 10.1016/j.chom.2017.02.003

[6] Gardiner CM , Mills KH. The cells that mediate innate immune memory and their functional significance in inflammatory and infectious diseases. Semin Immunol 2016; 28:343–50. 26979658 10.1016/j.smim.2016.03.001

[7] Naik S , Larsen SB , Gomez NC *et al* Inflammatory memory sensitizes skin epithelial stem cells to tissue damage. Nature 2017; 550:475–80. 29045388 10.1038/nature24271 PMC5808576

[8] Dai X , Medzhitov R. Inflammation: memory beyond immunity. Nature 2017; 550:460–1. 29045392 10.1038/nature24154

[9] Pate M , Damarla V , Chi DS , Negi S , Krishnaswamy G. Endothelial cell biology: role in the inflammatory response. Adv Clin Chem 2010; 52:109–30. 21275341

[10] Hall SE , Savill JS , Henson PM , Haslett C. Apoptotic neutrophils are phagocytosed by fibroblasts with participation of the fibroblast vitronectin receptor and involvement of a mannose/fucose‐specific lectin. J Immunol 1994; 153:3218–27. 7522254

[11] Boots AM , Wimmers‐Bertens AJ , Rijnders AW. Antigen‐presenting capacity of rheumatoid synovial fibroblasts. Immunology 1994; 82:268–74. 7927499 PMC1414827

[12] McGettrick HM , Butler LM , Buckley CD , Rainger GE , Nash GB. Tissue stroma as a regulator of leukocyte recruitment in inflammation. J Leukoc Biol 2012; 91:385–400. 22227966 10.1189/jlb.0911458

[13] McGettrick HM , Smith E , Filer A *et al* Fibroblasts from different sites may promote or inhibit recruitment of flowing lymphocytes by endothelial cells. Eur J Immunol 2009; 39:113–25. 19130557 10.1002/eji.200838232 PMC2821685

[14] Filer A , Ward LSC , Kemble S *et al* Identification of a transitional fibroblast function in very early rheumatoid arthritis. Ann Rheum Dis 2017; 76:2105–12. 28847766 10.1136/annrheumdis-2017-211286 PMC5705853

[15] Van Linthout S , Miteva K , Tschöpe C. Crosstalk between fibroblasts and inflammatory cells. Cardiovasc Res 2014; 102:258–69. 24728497 10.1093/cvr/cvu062

[16] Mor A , Abramson SB , Pillinger MH. The fibroblast‐like synovial cell in rheumatoid arthritis: a key player in inflammation and joint destruction. Clin Immunol 2005; 115:118–28. 15885632 10.1016/j.clim.2004.12.009

[17] Kaplanski G , Marin V , Montero‐Julian F , Mantovani A , Farnarier C. IL‐6: a regulator of the transition from neutrophil to monocyte recruitment during inflammation. Trends Immunol 2003; 24:25–9. 12495721 10.1016/s1471-4906(02)00013-3

[18] Navarro‐Xavier RA , Newson J , Silveira VL , Farrow SN , Gilroy DW , Bystrom J. A new strategy for the identification of novel molecules with targeted proresolution of inflammation properties. J Immunol 2010; 184:1516–25. 20032295 10.4049/jimmunol.0902866

[19] Greenlee‐Wacker MC. Clearance of apoptotic neutrophils and resolution of inflammation. Immunol Rev 2016; 273:357–70. 27558346 10.1111/imr.12453 PMC5000862

[20] Beeson PB , Roberts TAoE. Tolerance to bacterial pyrogens: II. Role of the reticulo‐endothelial system. J Exp Med 1947; 86:39–44. 19871653 10.1084/jem.86.1.39 PMC2135750

[21] Hollingsworth JW , Atkins E. Synovial inflammatory response to bacterial endotoxin. Yale J Biol Med 1965; 38:241–56. 5866671 PMC2591166

[22] Foster SL , Hargreaves DC , Medzhitov R. Gene‐specific control of inflammation by TLR‐induced chromatin modifications. Nature 2007; 447:972–8. 17538624 10.1038/nature05836

[23] Hamers L , Kox M , Pickkers P. Sepsis‐induced immunoparalysis: mechanisms, markers, and treatment options. Minerva Anestesiol 2015; 81:426–39. 24878876

[24] Tamura M , Tokuda M , Nagaoka S , Takada H. Lipopolysaccharides of *Bacteroides intermedius* (*Prevotella intermedia*) and *Bacteroides* (*Porphyromonas*) *gingivalis* induce interleukin‐8 gene expression in human gingival fibroblast cultures. Infect Immun 1992; 60:4932–7. 1328062 10.1128/iai.60.11.4932-4937.1992 PMC258250

[25] Lee TH , Lee GW , Ziff EB , Vilcek J. Isolation and characterization of eight tumor necrosis factor‐induced gene sequences from human fibroblasts. Mol Cell Biol 1990; 10:1982–8. 2183014 10.1128/mcb.10.5.1982-1988.1990 PMC360544

[26] Sakuta T , Tokuda M , Tamura M *et al* Dual regulatory effects of interferon‐alpha, ‐beta, and ‐gamma on interleukin‐8 gene expression by human gingival fibroblasts in culture upon stimulation with lipopolysaccharide from *Prevotella intermedia*, interleukin‐1alpha, or tumor necrosis factor‐alpha. J Dent Res 1998; 77:1597–605. 9719033 10.1177/00220345980770080701

[27] Wolchok JD , Vilcek J. Induction of HLA class I mRNA by cytokines in human fibroblasts: comparison of TNF, IL‐1 and IFN‐beta. Cytokine 1992; 4:520–7. 1292634 10.1016/1043-4666(92)90014-i

[28] Oliveira IC , Sciavolino PJ , Lee TH , Vilcek J. Downregulation of interleukin 8 gene expression in human fibroblasts: unique mechanism of transcriptional inhibition by interferon. Proc Natl Acad Sci USA 1992; 89:9049–53. 1409601 10.1073/pnas.89.19.9049 PMC50062

[29] Ospelt C , Reedquist KA , Gay S , Tak PP. Inflammatory memories: is epigenetics the missing link to persistent stromal cell activation in rheumatoid arthritis? Autoimmun Rev 2011; 10:519–24. 21497675 10.1016/j.autrev.2011.04.001

[30] Owens BM. Inflammation, innate immunity, and the intestinal stromal cell niche: opportunities and challenges. Front Immunol 2015; 6:319. 26150817 10.3389/fimmu.2015.00319 PMC4471728

[31] Dakin SG , Martinez FO , Yapp C *et al* Inflammation activation and resolution in human tendon disease. Sci Transl Med 2015; 7:311ra173. 10.1126/scitranslmed.aac4269 PMC4883654 26511510

[32] Ara T , Kurata K , Hirai K *et al* Human gingival fibroblasts are critical in sustaining inflammation in periodontal disease. J Periodontal Res 2009; 44:21–7. 19515019 10.1111/j.1600-0765.2007.01041.x

[33] Zaric SS , Coulter WA , Shelburne CE *et al* Altered Toll‐like receptor 2‐mediated endotoxin tolerance is related to diminished interferon beta production. J Biol Chem 2011; 286:29492–500. 21705332 10.1074/jbc.M111.252791 PMC3190989

[34] Sohn C , Lee A , Qiao Y , Loupasakis K , Ivashkiv LB , Kalliolias GD. Prolonged tumor necrosis factor α primes fibroblast‐like synoviocytes in a gene‐specific manner by altering chromatin. Arthritis Rheumatol 2015; 67:86–95. 25199798 10.1002/art.38871 PMC4455921

[35] Dakin SG , Newton J , Martinez FO *et al* Chronic inflammation is a feature of Achilles tendinopathy and rupture. Br J Sports Med 2017; 52: 359–367. 29118051 10.1136/bjsports-2017-098161 PMC5867427

[36] Dakin SG , Buckley CD , Al‐Mossawi MH *et al* Persistent stromal fibroblast activation is present in chronic tendinopathy. Arthritis Res Ther 2017; 19:16. 28122639 10.1186/s13075-016-1218-4 PMC5264298

[37] Crowley T , O'Neil JD , Adams H *et al* Priming in response to pro‐inflammatory cytokines is a feature of adult synovial but not dermal fibroblasts. Arthritis Res Ther 2017; 19:35. 28187781 10.1186/s13075-017-1248-6 PMC5303242

[38] Klein K , Frank‐Bertoncelj M , Karouzakis E *et al* The epigenetic architecture at gene promoters determines cell type‐specific LPS tolerance. J Autoimmun 2017; 83:122–33. 28701277 10.1016/j.jaut.2017.07.001

[39] Sohn C , Lee A , Qiao Y , Loupasakis K , Ivashkiv LB , Kalliolias GD. Prolonged TNFα primes fibroblast‐like synoviocytes in a gene‐specific manner by altering chromatin. Arthritis Rheumatol 2014; 67:86–95. 10.1002/art.38871 PMC4455921 25199798

[40] Silverman MD , Tumuluri RJ , Davis M , Lopez G , Rosenbaum JT , Lelkes PI. Homocysteine upregulates vascular cell adhesion molecule‐1 expression in cultured human aortic endothelial cells and enhances monocyte adhesion. Arterioscler Thromb Vasc Biol 2002; 22:587–92. 11950695 10.1161/01.atv.0000014221.30108.08

[41] Wang W , Deng M , Liu X , Ai W , Tang Q , Hu J. TLR4 activation induces nontolerant inflammatory response in endothelial cells. Inflammation 2011; 34:509–18. 20878353 10.1007/s10753-010-9258-4

[42] Koch SR , Lamb FS , Hellman J , Sherwood ER , Stark RJ. Potentiation and tolerance of toll‐like receptor priming in human endothelial cells. Transl Res 2017; 180:53–67.e4. 27567430 10.1016/j.trsl.2016.08.001 PMC5253081

[43] Chaudhury H , Zakkar M , Boyle J *et al* c‐Jun N‐terminal kinase primes endothelial cells at atheroprone sites for apoptosis. Arterioscler Thromb Vasc Biol 2010; 30:546–53. 20056910 10.1161/ATVBAHA.109.201368

[44] Zakkar M , Chaudhury H , Sandvik G *et al* Increased endothelial mitogen‐activated protein kinase phosphatase‐1 expression suppresses proinflammatory activation at sites that are resistant to atherosclerosis. Circ Res 2008; 103:726–32. 18723442 10.1161/CIRCRESAHA.108.183913

[45] Oliveira SD , Quintas LE , Amaral LS , Noël F , Farsky SH , Silva CL. Increased endothelial cell‐leukocyte interaction in murine schistosomiasis: possible priming of endothelial cells by the disease. PLOS ONE 2011; 6:e23547. 21853150 10.1371/journal.pone.0023547 PMC3154496

[46] Séguin C , Abid MR , Spokes KC *et al* Priming effect of homocysteine on inducible vascular cell adhesion molecule‐1 expression in endothelial cells. Biomed Pharmacother 2008; 62:395–400. 18406566 10.1016/j.biopha.2008.02.008 PMC5378488

[47] Zemani F , Silvestre JS , Fauvel‐Lafeve F *et al* *Ex vivo* priming of endothelial progenitor cells with SDF‐1 before transplantation could increase their proangiogenic potential. Arterioscler Thromb Vasc Biol 2008; 28:644–50. 18239152 10.1161/ATVBAHA.107.160044

[48] Stark RJ , Choi H , Koch SR , Fensterheim BA , Lamb FS , Sherwood ER. Endothelial cell tolerance to lipopolysaccharide challenge is induced by monophosphoryl lipid A. Clin Sci (Lond) 2016; 130:451–61. 26669797 10.1042/CS20150592 PMC4744094

[49] Seeley JJ , Ghosh S. Molecular mechanisms of innate memory and tolerance to LPS. J Leukoc Biol 2017; 101:107–19. 27780875 10.1189/jlb.3MR0316-118RR

[50] Kawasaki T , Kawai T. Toll‐like receptor signaling pathways. Front Immunol 2014; 5:461. 25309543 10.3389/fimmu.2014.00461 PMC4174766

[51] Serhan CN , Savill J. Resolution of inflammation: the beginning programs the end. Nat Immunol 2005; 6:1191–7. 16369558 10.1038/ni1276

[52] Murray PJ , Smale ST. Restraint of inflammatory signaling by interdependent strata of negative regulatory pathways. Nat Immunol 2012; 13:916–24. 22990889 10.1038/ni.2391 PMC4893774

[53] Kobayashi K , Hernandez LD , Galán JE , Janeway CA , Medzhitov R , Flavell RA. IRAK‐M is a negative regulator of Toll‐like receptor signaling. Cell 2002; 110:191–202. 12150927 10.1016/s0092-8674(02)00827-9

[54] Zou XL , Pei DA , Yan JZ , Xu G , Wu P. A20 overexpression inhibits lipopolysaccharide‐induced NF‐κB activation, TRAF6 and CD40 expression in rat peritoneal mesothelial cells. Int J Mol Sci 2014; 15:6592–608. 24747594 10.3390/ijms15046592 PMC4013649

[55] Cekic C , Casella CR , Sag D *et al* MyD88‐dependent SHIP1 regulates proinflammatory signaling pathways in dendritic cells after monophosphoryl lipid A stimulation of TLR4. J Immunol 2011; 186:3858–65. 21339365 10.4049/jimmunol.1001034 PMC3249415

[56] Porta C , Rimoldi M , Raes G *et al* Tolerance and M2 (alternative) macrophage polarization are related processes orchestrated by p50 nuclear factor kappaB. Proc Natl Acad Sci USA 2009; 106:14978–83. 19706447 10.1073/pnas.0809784106 PMC2736429

[57] Lee A , Qiao Y , Grigoriev G *et al* Tumor necrosis factor α induces sustained signaling and a prolonged and unremitting inflammatory response in rheumatoid arthritis synovial fibroblasts. Arthritis Rheum 2013; 65:928–38. 23335080 10.1002/art.37853 PMC3618592

[58] Xiong Y , Medvedev AE. Induction of endotoxin tolerance *in vivo* inhibits activation of IRAK4 and increases negative regulators IRAK‐M, SHIP‐1, and A20. J Leukoc Biol 2011; 90:1141–8. 21934070 10.1189/jlb.0611273 PMC3236548

[59] Chen J , Ivashkiv LB. IFN‐γ abrogates endotoxin tolerance by facilitating Toll‐like receptor‐induced chromatin remodeling. Proc Natl Acad Sci USA 2010; 107:19438–43. 20974955 10.1073/pnas.1007816107 PMC2984206

[60] Rosengren S , Corr M , Firestein GS , Boyle DL. The JAK inhibitor CP‐690,550 (tofacitinib) inhibits TNF‐induced chemokine expression in fibroblast‐like synoviocytes: autocrine role of type I interferon. Ann Rheum Dis 2012; 71:440–7. 22121136 10.1136/ard.2011.150284

[61] Chen X , Yoza BK , El Gazzar M , Hu JY , Cousart SL , McCall CE. RelB sustains IkappaBalpha expression during endotoxin tolerance. Clin Vaccine Immunol 2009; 16:104–10. 19020113 10.1128/CVI.00320-08 PMC2620660

[62] Arts RJ , Carvalho A , La Rocca C *et al* Immunometabolic pathways in BCG‐induced trained immunity. Cell Rep 2016; 17:2562–71. 27926861 10.1016/j.celrep.2016.11.011 PMC5177620

[63] del Fresno C , García‐Rio F , Gómez‐Piña V *et al* Potent phagocytic activity with impaired antigen presentation identifying lipopolysaccharide‐tolerant human monocytes: demonstration in isolated monocytes from cystic fibrosis patients. J Immunol 2009; 182:6494–507. 19414804 10.4049/jimmunol.0803350

[64] El Gazzar M. HMGB1 modulates inflammatory responses in LPS‐activated macrophages. Inflamm Res 2007; 56:162–7. 17522814 10.1007/s00011-006-6112-0

[65] Ostuni R , Piccolo V , Barozzi I *et al* Latent enhancers activated by stimulation in differentiated cells. Cell 2013; 152:157–71. 23332752 10.1016/j.cell.2012.12.018

[66] Baker B , Maitra U , Geng S , Li L. Molecular and cellular mechanisms responsible for cellular stress and low‐grade inflammation induced by a super‐low dose of endotoxin. J Biol Chem 2014; 289:16262–9. 24759105 10.1074/jbc.M114.569210 PMC4047395

[67] Loppnow H , Werdan K , Buerke M. Vascular cells contribute to atherosclerosis by cytokine‐ and innate‐immunity‐related inflammatory mechanisms. Innate Immun 2008; 14:63–87. 18713724 10.1177/1753425908091246

[68] Bekkering S , Joosten LA , van der Meer JW , Netea MG , Riksen NP. Trained innate immunity and atherosclerosis. Curr Opin Lipidol 2013; 24:487–92. 24184939 10.1097/MOL.0000000000000023

[69] Bekkering S , Joosten LA , van der Meer JW , Netea MG , Riksen NP. The epigenetic memory of monocytes and macrophages as a novel drug target in atherosclerosis. Clin Ther 2015; 37:914–23. 25704108 10.1016/j.clinthera.2015.01.008

[70] Sitia S , Tomasoni L , Atzeni F *et al* From endothelial dysfunction to atherosclerosis. Autoimmun Rev 2010; 9:830–4. 20678595 10.1016/j.autrev.2010.07.016

[71] Bottini N , Firestein GS. Duality of fibroblast‐like synoviocytes in RA: passive responders and imprinted aggressors. Nat Rev Rheumatol 2013; 9:24–33. 23147896 10.1038/nrrheum.2012.190 PMC3970924

[72] Patel R , Filer A , Barone F , Buckley CD. Stroma: fertile soil for inflammation. Best Pract Res Clin Rheumatol 2014; 28:565–76. 25481550 10.1016/j.berh.2014.10.022

[73] Parsonage G , Filer AD , Haworth O *et al* A stromal address code defined by fibroblasts. Trends Immunol 2005; 26:150–6. 15745857 10.1016/j.it.2004.11.014 PMC3121558

[74] Didierlaurent A , Goulding J , Hussell T. The impact of successive infections on the lung microenvironment. Immunology 2007; 122:457–65. 17991012 10.1111/j.1365-2567.2007.02729.x PMC2266032

[75] Goulding J , Snelgrove R , Saldana J *et al* Respiratory infections: do we ever recover? Proc Am Thorac Soc 2007; 4:618–25. 18073393 10.1513/pats.200706-066TH PMC2647650

[76] Kaynar AM , Yende S , Zhu L *et al* Effects of intra‐abdominal sepsis on atherosclerosis in mice. Crit Care 2014; 18:469. 25182529 10.1186/s13054-014-0469-1 PMC4172909

[77] Yende S , D'Angelo G , Mayr F *et al* Elevated hemostasis markers after pneumonia increases one‐year risk of all‐cause and cardiovascular deaths. PLOS ONE 2011; 6:e22847. 21853050 10.1371/journal.pone.0022847 PMC3154260

[78] Thøstesen LM , Nissen TN , Kjærgaard J *et al* Bacillus Calmette–Guérin immunisation at birth and morbidity among Danish children: a prospective, randomised, clinical trial. Contemp Clin Trials 2015; 42:213–8. 25896113 10.1016/j.cct.2015.04.006

[79] Karumuthil‐Melethil S , Gudi R , Johnson BM , Perez N , Vasu C. Fungal β‐glucan, a Dectin‐1 ligand, promotes protection from type 1 diabetes by inducing regulatory innate immune response. J Immunol 2014; 193:3308–21. 25143443 10.4049/jimmunol.1400186 PMC4170060

[80] Arens C , Bajwa SA , Koch C *et al* Sepsis‐induced long‐term immune paralysis–results of a descriptive, explorative study. Crit Care 2016; 20:93. 27056672 10.1186/s13054-016-1233-5 PMC4823837

[81] Demmer RT , Papapanou PN. Epidemiologic patterns of chronic and aggressive periodontitis. Periodontol 2000 2010; 53:28–44. 20403103 10.1111/j.1600-0757.2009.00326.x PMC3406186

[82] Vanniasinghe AS , Manolios N , Schibeci S *et al* Targeting fibroblast‐like synovial cells at sites of inflammation with peptide targeted liposomes results in inhibition of experimental arthritis. Clin Immunol 2014; 151:43–54. 24513809 10.1016/j.clim.2014.01.005

[83] Müller‐Ladner U , Kriegsmann J , Franklin BN *et al* Synovial fibroblasts of patients with rheumatoid arthritis attach to and invade normal human cartilage when engrafted into SCID mice. Am J Pathol 1996; 149:1607–15. 8909250 PMC1865262

[84] Ghesquière B , Wong BW , Kuchnio A , Carmeliet P. Metabolism of stromal and immune cells in health and disease. Nature 2014; 511:167–76. 25008522 10.1038/nature13312

[85] Naughton DP , Haywood R , Blake DR , Edmonds S , Hawkes GE , Grootveld M. A comparative evaluation of the metabolic profiles of normal and inflammatory knee‐joint synovial fluids by high resolution proton NMR spectroscopy. FEBS Lett 1993; 332:221–5. 7691662 10.1016/0014-5793(93)80636-9

[86] Chimenti MS , Triggianese P , Conigliaro P , Candi E , Melino G , Perricone R. The interplay between inflammation and metabolism in rheumatoid arthritis. Cell Death Dis 2015; 6:e1887. 26379192 10.1038/cddis.2015.246 PMC4650442

[87] Cheng SC , Quintin J , Cramer RA *et al* mTOR‐ and HIF‐1α‐mediated aerobic glycolysis as metabolic basis for trained immunity. Science 2014; 345:1250684. 25258083 10.1126/science.1250684 PMC4226238

[88] Rinn JL , Bondre C , Gladstone HB , Brown PO , Chang HY. Anatomic demarcation by positional variation in fibroblast gene expression programs. PLOS Genet 2006; 2:e119. 16895450 10.1371/journal.pgen.0020119 PMC1523235

[89] Filer A , Antczak P , Parsonage GN *et al* Stromal transcriptional profiles reveal hierarchies of anatomical site, serum response and disease and identify disease specific pathways. PLOS ONE 2015; 10:e0120917. 25807374 10.1371/journal.pone.0120917 PMC4373951

[90] Parsonage G , Filer A , Bik M *et al* Prolonged, granulocyte‐macrophage colony‐stimulating factor‐dependent, neutrophil survival following rheumatoid synovial fibroblast activation by IL‐17 and TNFalpha. Arthritis Res Ther 2008; 10:R47. 18433499 10.1186/ar2406 PMC2453767

[91] Filer A , Parsonage G , Smith E *et al* Differential survival of leukocyte subsets mediated by synovial, bone marrow, and skin fibroblasts: site‐specific versus activation‐dependent survival of T cells and neutrophils. Arthritis Rheum 2006; 54:2096–108. 16802344 10.1002/art.21930 PMC3119431

